# Characteristics of unintentional drowning deaths in children with autism spectrum disorder

**DOI:** 10.1186/s40621-017-0129-4

**Published:** 2017-12-08

**Authors:** Joseph Guan, Guohua Li

**Affiliations:** 1 Department of Epidemiology, Columbia University Mailman School of Public Health, DrPH; 622 West 168th St, New York, NY PH5-505 USA; 2 Department of Anesthesiology, Columbia University College of Physicians and Surgeons, New York, NY USA; 3 Center for Injury Epidemiology and Prevention, Columbia University Medical Center, New York, NY USA

**Keywords:** Accidental death, Autism, Drowning, Epidemiology, Injury, Wandering

## Abstract

**Background:**

The reported prevalence of autism spectrum disorder (ASD) has increased markedly in the past two decades. Recent research indicates that children with ASD are at a substantially increased risk of injury mortality, particularly from unintentional drowning. The purpose of this study was to explore the circumstances of fatal unintentional drowning incidents involving children with ASD under 15 years of age.

**Findings:**

During January 2000 through May 2017, US newspapers reported a total of 23 fatal drowning incidents involving 18 boys and 5 girls with ASD. Age of victims ranged from 3 to 14 years (mean = 7.7 ± 2.9 years). These drowning incidents most commonly occurred in ponds (52.2%), followed by rivers (13.0%), and lakes (13.0%). For 11 incidents with location data available, the distance between victim residence and the water body where drowning occurred averaged 290.7 m (± 231.5 m). About three-quarters (73.3%) of the drowning incidents occurred in the afternoon hours from 12:00 to 18:59. Wandering was the most commonly reported activity that led to drowning, accounting for 73.9% of the incidents.

**Conclusions:**

Fatal drowning in children with ASD typically occur in water bodies near the victims’ homes in the afternoon hours precipitated by wandering. Multifaceted intervention programs are urgently needed to reduce the excess risk of drowning in children with ASD.

## Background

Autism spectrum disorder (ASD) is a complex neurodevelopmental disability characterized by repetitive behaviors and impairments in social and communication skills (American Psychiatric Association, 2013). Symptoms may emerge in early childhood, usually beginning at age 2 to 3 years, and last throughout adulthood (American Psychiatric Association, 2013; National Institute of Mental Health, 2016; Perou et al., 2013). Some people with ASD demonstrate maladaptive behaviors including self-harm, wandering, aggression, and uncontrolled outbursts from anger or irritation (Carroll et al., 2014; Green et al., 2000; Kanne & Mazurek, 2011). Autism can co-occur with other medical and developmental conditions (e.g. epilepsy and intellectual disability) and symptoms that pose potential challenges to everyday functioning (American Psychiatric Association, 2013).

The reported prevalence of ASD has increased considerably over the past two decades, from 1 in 150 children to about 1 in 68 children (Centers for Disease Control and Prevention, 2017; Christensen et al., 2016). Currently, there is an estimated 3.5 million people living with ASD in the United States, including approximately 850,000 children under the age of 17 years (Buescher et al., 2014).

Although little is known about the long-term health outcomes of people with ASD, research indicates that individuals with this disorder are at up to 10-fold increased risk of premature death compared to the general population (Rice et al., 2016; Gillberg et al., 2010; Mouridsen et al., 2008; Pickett et al., 2011). A recent study provided compelling evidence that compared to the general pediatric population, children with ASD are at a substantially heightened risk of unintentional injury, in particularly drowning (Guan & Li, 2017). Although drowning remains a major cause of childhood mortality worldwide, the circumstances preceding the incidents are often understudied or undocumented in death records (World Health Organization Global Report on Drowning, 2014). In order to develop targeted strategies to reduce the excess risk of drowning, it is imperative to understand the circumstances of the drowning incidents. Thus, the purpose of this study was to examine the frequency and characteristics of fatal unintentional drowning incidents involving children with ASD.

## Methods

The Lexis-Nexis^®^ Academic database is a web-based research database that contains archives of full-text documents from over 17,000 sources including newspapers, journals, wire services, business and company publications, and legal government documents. The database was queried using terms ((‘autism’ OR ‘autistic’) AND (‘drowning’ or ‘drowned’) AND (‘boy’ or ‘girl’)) that appeared in the headline or body of the newspaper articles to identify the drowning incidents involving children under age 15 years with ASD in the United States from January 2000 through May 2017. Duplicate options were set for ‘On-high similarity’.

All items returned from the query were screened and information related to the victims and details of the incidents, including time of day and distance from residence, were ascertained from the newspaper reports and analyzed using descriptive statistics.

## Results

**Fig. 1:**
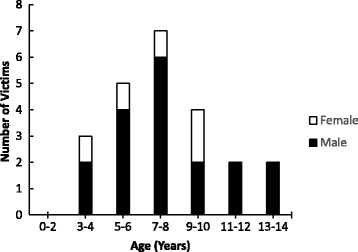
Age Distribution of Fatal Unintentional Drowning Incidents Involving Children with Autism Spectrum Disorder by Sex as Reported in US Newspapers, January 2000 – May 2017

**Fig. 2:**
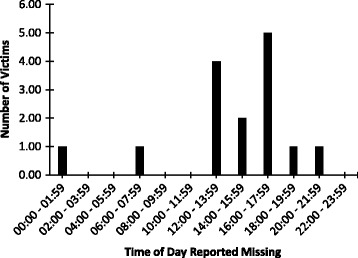
Time of Day Distribution of Fatal Unintentional Drowning Incidents Involving Children with Autism Spectrum Disorder as Reported in US Newspapers, January 2000 – May 2017

During January 2000 through May 2017, US newspapers reported a total of 23 fatal unintentional drowning incidents involving children under 15 years of age with ASD. Of the 23 incidents, 14 (60.9%) occurred between 2010 and 2016. There was no incident reported in 2017 as of May 31st, 2017. The majority (78.3%) of the victims were boys. Age of drowning victims ranged from 3 to 14 years (mean = 7.7 ± 2.9 years), with female victims being 1.1 years younger on average than male victims (Fig. 1). The most common water body where the fatal drowning incidents occurred was ponds (52.2%), followed by lakes (13.0%), and rivers (13.0%) (Table 1). Data about proximity of the water body to the victim’s residence were available for 11 (47.8%) of the incidents, with all of them within 1000 m of the victim’s residence (mean = 290.7 ± 231.5 m). The time of day at which victims were reported missing was available for 15 (65.2%) of 23 incidents, with 2 (13.3%) being in the morning (0:00–11:59), 11 (73.3%) being in the afternoon (12:00–17:59), and 2 (13.3%) being in the evening (18:00 PM – 23:59) (Fig. 2). The drowning incidents occurred most frequently in the month of June (21.7%). Wandering was the most commonly reported activity that led to drowning, accounting for 73.9% of the incidents (Table 1).

**Table 1:** Circumstances of Fatal Accidental Drowning Incidents Involving Children with Autism Spectrum Disorder as Reported in US Newspapers, January 2000 – May 2017

Circumstance	*n* (%)
Water body type	
Pond	12 (52.2)
Lake	3 (13.0)
River	3 (13.0)
Creek	2 (8.7)
Private pool	1 (4.3)
Public pool	1 (4.3)
Bathtub	1 (4.3)
Activity leading to drowning	
Wandering	17 (73.9)
Left unattended to	1 (4.3)
Unspecified	5 (21.7)

## Discussion

The results of this study indicate that fatal unintentional drownings involving children with ASD reported in US newspapers have increased considerably since 2010. One plausible explanation for this increase might be the increased prevalence and awareness of ASD (Guan & Li, 2017). Furthermore, the results indicate that fatal unintentional drowning in children with ASD typically involved boys aged 7–8 years.

Our results also show that most of the victims wandered away from their residence and drowned in nearby ponds. Previous studies have suggested that wandering behavior is highest among children with ASD and intellectual disability, especially among boys between 6 and 11 years of age (Rice et al., 2016; Kiely et al., 2016; Barnard-Brak et al., 2016; Anderson et al., 2012). One often cited contributing factor for child drowning is a lapse in or lack of caregiver supervision (Blum & Shield, 2000; Hyder et al., 2009). Therefore, autistic children showing the tendency of wandering may benefit from real-time monitoring through global positioning system devices. Drowning risk assessment (e.g., proximity of water bodies) and risk reduction in the residential environment (e.g., barriers to water bodies) should also be considered. In residential environments for children with ASD, it is desirable to have devices that quickly alert parents and guardians when the children elope from the premises.

According to the narratives provided in the news reports, many of the drowning victims were fascinated with water and drowned in “unfenced” or “unguarded” ponds. While it is necessary to mitigate the behavioral and environmental risks by restricting access into water bodies through secured barriers or fences (Leavy et al., 2016; Wallis et al., 2015), parents, pediatricians, and other caretakers should help provide swimming lessons to these children as soon as the diagnosis is made (Guan & Li, 2017). Studies have shown that children who receive swimming and water safety (e.g., survival flotation, energy conservation, and safety behavior) lessons at an earlier age will acquire the skills faster and thus may improve their survival chances in dangerous situations (Wallis et al., 2015). It is feasible and effective to improve water safety skills in children with ASD through aquatic training (Alaniz et al., 2017). In addition, parents should be encouraged to receive water safety training, which may help reduce the drowning risk for their children (Leavy et al., 2016).

This study is limited by the small sample size and the availability of information reported in newspaper articles. Often, basic demographic data and other important details are missed in newspaper reports (Baullinger et al., 2001; Voight et al., 1998). As a resource for sentinel injury surveillance, newspaper reports are likely to uncover only the tip of the “iceberg.” In order to have a more complete understanding and develop interventions to reduce the excess risk of unintentional drowning involving children with ASD, future studies should use more rigorously collected data from injury surveillance systems.

## Conclusions

Findings from this study indicate that fatal unintentional drownings involving children with ASD reported in US newspapers have increased in the recent years. These incidents occur commonly in unsecured ponds, rivers, and lakes that are in near proximity to the victims’ residence. Furthermore, fatal unintentional drownings in children with ASD typically involve boys ages 7–8 years who wander away from home to nearby ponds in the afternoon hours. Given these findings, intervention programs for children with ASD should be developed to prevent these children from getting into bodies of water unsupervised. Furthermore, to reduce the excess risk of drowning, children with ASD should be provided with swimming lessons and water safety training immediately following diagnosis.
